# MIR4435-2HG in exosomes promotes gastric carcinogenesis by inducing M2 polarization in macrophages

**DOI:** 10.3389/fonc.2022.1017745

**Published:** 2022-11-22

**Authors:** Chaofeng Li, Zhengju Chen, Jinli Gao, Tao Tang, Lei Zhou, Guochao Zhang, Dongdong Zhang, Chao Shen, Lei Guo, Tao Fu

**Affiliations:** ^1^ Gastrointestinal Surgery Department, China-Japan Friendship Hospital, Beijing, China; ^2^ Nanchang Institute of Technology, College of Medicine, China. Pooling Medical Research Institutes, Hangzhou, China; ^3^ Pooling Medical Research Institutes, Hangzhou, Beijing, China; ^4^ Department of Pathology, East Hospital, Tongji University, Shanghai, China; ^5^ Gastrointestinal Surgery, Peking University International Hospital, Beijing, China; ^6^ Department of Gastrointestinal Surgery, Key Laboratory of Carcinogenesis and Translational Research (Ministry of Education), Peking University Cancer Hospital and Institute, Beijing, China

**Keywords:** GC, MKN45, MIR4435-2HG, Jagged1/Notch, JAK1/STAT3

## Abstract

Gastric cancer (GC) is a cancer with a high mortality rate. lncRNAs play a role in regulating GC tumorigenesis. In this paper, we analyzed differentially expressed lncRNAs between GC and adjacent normal tissues using multiple bioinformatics tools to identify new potential targets in GC. Cell viability and migration ability were detected using the Cell Counting Kit-8 (CCK-8) and transwell assays, MIR4435-2HG was negatively correlated with the survival rate of GC patients, and by inhibiting the activity of MIR4435-2HG, the viability and migration ability of GC cells could be reduced. In addition, RT- qPCR and western blot to detect gene and protein level expression, transmission electron microscopy and nanoparticle tracking analysis (NTA) to study the efficiency of exosome isolation, and flow cytometry to observe cell differentiation were employed, delivery of MIR4435-2HG shRNA *via* MKN45 cell-derived exosomes significantly reversed the MKN45 exosome-induced M2 polarization in macrophages. Furthermore, the low expression of MIR4435-2HG in MKN45 cell-derived exosomes inhibited the Jagged1/Notch and JAK1/STAT3 pathways in macrophages; MIR4435-2HG downregulated exosomes were found to significantly inhibit GC tumor growth *in vivo* by establishing a mouse model. In short, MKN45 cell-derived exosomes deliver lncRNA MIR4435-2HG, which promotes gastric carcinogenesis by inducing macrophage M2 polarization.

## Introduction

Gastric cancer is one of the most common high-mortality cancers. Accounting for 8% of all cancer types in terms of both cancer incidence and mortality (1, 2). The main cause of its poor prognosis is distant metastasis, which accounts for approximately 90% of cancer-related deaths (3). Surgical resection with or without chemotherapy is usually used clinically for GC, but treatment outcomes have not met expectations so far (4). Therefore, it is an important and urgent matter to find the pathogenesis of GC-related molecules and promote targeted therapy.

Long-stranded noncoding RNAs are noncoding RNAs greater than 200 bp in length with limited protein-coding potential (5), and several existing findings have shown that lncRNAs play a role in epigenetic, transcriptional, and posttranscriptional levels, as well as in cancer development (6, 7), and lncRNAs play a key role in gastric carcinogenesis (8, 9).

Exosomes are a type of vesicle secreted from the mammalian intracellular to the extracellular, consisting of membranes of multivesicular bodies, which can participate in cellular communication by transferring proteins and nucleic acids (10–13) and are an efficient drug carrier in clinical applications (14). Exosomes can play a role in tumorigenesis through communication with tumor cells and macrophages and exhibit immunosuppressive effects to some extent (15, 16). In contrast, macrophages (especially M2 macrophages) can suppress the immune response in tumor development and thus can promote drug resistance in cancer therapy (17, 18), which is widely used in clinical practice. Small interfering RNA (siRNA) is a therapeutic agent that can treat a variety of diseases, but safe, efficient, and targeted delivery of siRNA is still one of the main challenges (19). This paper focuses on exosome-targeted delivery of siRNA.

**Figure 1:**
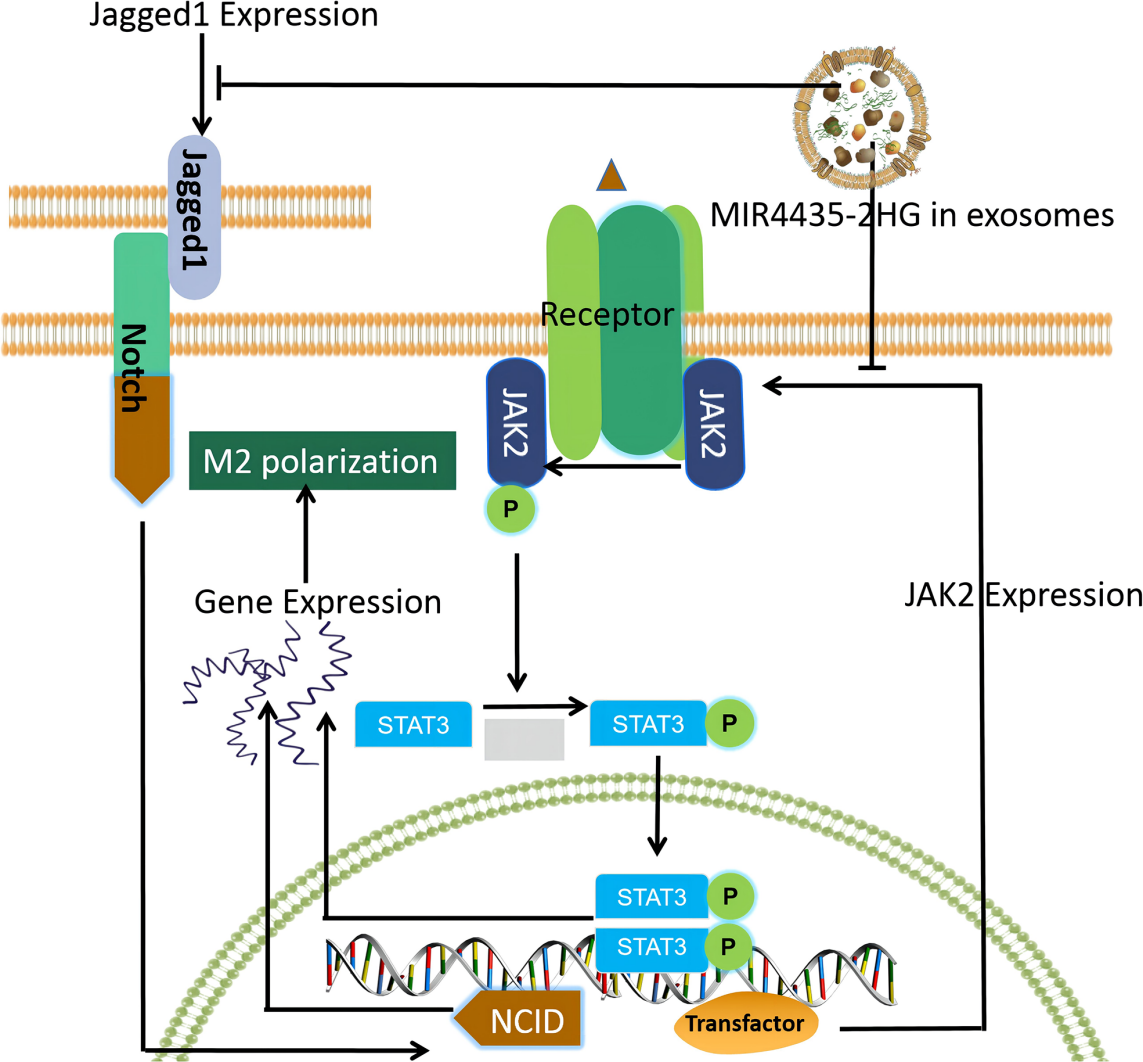
Macrophage M2 polarization pathway mapped based on our findings.

In this study, we investigated the differentially expressed lncRNAs between GC and adjacent normal tissues to find GC-associated lncRNAs and investigated the exosomal transport of siRNA, which could represent a breakthrough for GC clinical treatment and research. We found that MIR4435-2HG in exosomes is a key factor in regulating the promotion of macrophage M2 polarization and mapped the macrophage M2 polarization pathway based on this theory (**Figure 1**).

## Materials and methods

### GC lncRNA differential expression analysis

The GC lncRNA dataset was got from the Cancer Genome Atlas database, and differential analysis was performed using the R software limma package. The results are presented in volcano plots with a threshold of |log2FC| > 1, P < 0.05. The most significantly differentially expressed gene was selected as the key lncRNA. The R software ggplot2 package was employed to analyze the differential expression of this lncRNA in tumor nd normal samples. The results are illustrated by box plots. P < 0.05 was statistically significant.

### Cell culture

Human normal gastric epithelial cell lines (GES-1), GC cell lines (MKN-45AGS and SNU-5), macrophages (THP-1), and 293T cells were placed in DMEM solution (Thermo Fisher Scientific, Waltham, MA, USA) with 10% FBS and 1% penicillin and streptomycin and incubated at a constant temperature of 37°C.

### Cell transfection

MIR4435-2HG shRNA1 and shRNA2, nontargeting sequences from Hanbio Biotechnology Co., Ltd, and OE from Shanghai GenePharma Co., Ltd were packaged into lentiviral vectors. The lentiviral vector DNA was transfected into 293T cells and incubated at 37°C after transfection. The supernatant was filtered into pellets, and the lentiviral pellets were infected with GC cells. qPCR was used to verify the transfection efficiency.

### Extraction and identification of exosomes

Cells were first cultured in a complementary medium, and when the cells reach 80% fusion with the culture medium, the medium was replaced with an FBS-free medium for 2 days, the supernatant was collected by centrifugation and filtration (Millipore, USA), and the supernatant was collected by ultracentrifugation (Beckman Coulter) to obtain exosomes. The subgroups were named KN45-Exo-NC, MKN45-Exo-MIR4435-2HG OE or MKN45-Exo-MIR4435-2HGshRNA2, respectively. Exosomes were identified using transmission electron microscopy and immunoblotting.

### Macrophage isolation and culture

Macrophages were treated with 100 ng/ml of PMA (Peprotech) and then with PBS, MKN45-Exo-NC, MKN45-Exo-MIR4435-2HG OE, or MKN45-Exo-MIR4435-2HG shRNA2 for 24 h. Macrophages were isolated using a flow cytometer (New Jersey, USA) for the macrophage surface markers CD206 (eBioscience) and CD68 (eBioscience).

### Reverse transcription-quantitative PCR (RT-qPCR)

The total RNA of samples was extracted from cells using TRIzol reagents and reverse transcribed into cDNA using the PrimeScript RT kit, and qPCR was performed using the SYBR premix Ex Taq II kit to detect gene expression. The expression of MIR4435-2HG, arginase-1, and iNOS genes was measured using β-actin as an internal reference (**Table 1**). The relative expression of the genes was calculated using the 2-ΔΔCT method.

**Table 1:** Gene primer sequences.

Symbol	Forward	Reverse
MIR4435-2HG	5’-TGGGCAATAGGCGATACGAT-3’	5’-TGGGCAATAGGCGATACGAT-3’
Arginase-1	5’-CACCTGGGCAAGGATTCA-3’	5’-CTCAACTGGTGTCGTGGAGTC-3’
iNOS	5’-CTCAACTGGTGTCGTGGAGTC-3’	5’-ACAGCACACCGTAGTCTCGG-3’
β-actin	5’-GTCCACCGCAAATGCTTCTA-3’	5’-TGCTGTCACCTTCACCGTTC-3’

### Cell Counting Kit-8 (CCK-8) assay

GC cells (5 × 103 cells/well) were plated in 96-well plates, and NC, MIR4435-2HG shRNA2, and MIR4435-2HG OE were treated at 37°C with 5% CO2 for 0, 24, 48, and 72 h. Ten microliters of CCK-8 reagent was added and incubated at 37°C for 2 h. The absorbance of each well was measured at 450 nm using an enzyme marker (Thermo Fisher Scientific).

### Western blotting

Total proteins were extracted using RIPA lysate (Beyotime) and quantified using the bicinchoninic acid protein kit (Thermo Fisher Scientific). Proteins were separated by 10% SDS-PAGE, transferred to PVDF membranes, and blocked with 5% skim milk at room temperature for 1 h. The following primary antibodies were used with β-actin as an internal reference: CD63 (Abcam; ab134045), TSG101 (Abcam; ab125011), Notch1 (Abcam; ab52627), Notch2 (Abcam;ab118824), vimentin (CST; #5741), N-cadherin (CST; #13116), E-cadherin (CST; #14472), STAT3 (Abcam; ab68153), p-STAT3 (Abcam; ab267373), JAK1 (CST; #29261), p-JAK1 (CST; #3331), Jagged1 (CST; #2620), Hes1 (CST; #11988), Hes5 (Abcam; ab194111), and β-actin (Abcam; ab8226). After initial incubation, membranes were incubated with HRP-coupled secondary antibodies for 1 h at room temperature. Protein bands were visualized using an ECL kit. Densitometric analysis was performed using IPP6.0 (Image-ProPlus6.0).

### Immunofluorescence

Cells were inoculated into 24-well plates overnight, prefixed with 4% paraformaldehyde for 10 min, and then fixed in methanol for 10 min. Cells were stained with Phalloidin-iFluor 488 reagent (ab176753) and the PKH26 Red Cell Membrane Staining Kit (D0030, Solarbio), respectively. Phalloidin-iFluor 488 stains the cytoskeleton and PKH26 stains the cell membrane, enabling localization of the exosome.

### Cell migration assay

GC cells were inoculated in the upper chamber of the medium containing 1% FBS and the density was adjusted to approximately 1.0×10^6^ cells per chamber. RPMI 1640 medium containing 10% FBS was added to the lower chamber. After incubation at 37°C for 24 h, the transfer chamber was rinsed twice (5 min each time) with PBS. The cells were fixed with 5% glutaraldehyde at 4°C, stained with 0.1% crystal violet for 30 min, and the transwell chambers were washed twice with PBS, followed by observation under a microscope. The number of migrating cells was considered to be a reflection of migratory capacity.

### *In vivo* study

Thirty BALB/c nude mice (6-8 weeks old) purchased from VitalLiver (Beijing, China) were housed in a dedicated SPF facility for culturing. MKN45 cells were co-cultured with macrophages; macrophages-exo, macrophagesexo-NC, macrophagesexo-MIR4435-2HG OE, or macrophagesexo-MIR4435-2HG shRNA2 was transplanted into BALB/c nude mice, and tumor size was measured regularly.

### Statistical analysis

Three independent replicate experiments were performed for each group of samples, and the results are presented as mean ± standard deviation (SD). Statistical methods included the t-test (with 2 groups) and one-way ANOVA (with 3 or more groups), and Tukey’s test was used. P < 0.05 was statistically significant.

## Results

### GC lncRNA differential expression results

**Figure 2:**
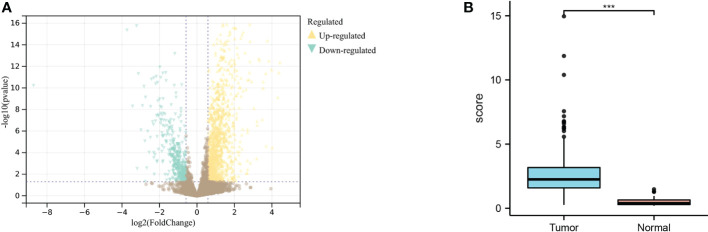
Differential analysis of TCGA dataset for gastric cancer. **(A)** Differential expression results of the TCGA dataset; yellow indicates upregulated genes, blue indicates downregulated genes. **(B)**. Analysis of MIR4435-2HG expression in gastric cancer tissues and normal tissues adjacent to cancer, blue indicates tumor, and red indicates normal tissue. *** indicates P<0.001.

Through differential analysis of GC and normal samples next to cancer, 847 genes were obtained, including 636 upregulated genes and 211 downregulated genes (**Figure 2A**). The expression results of MIR4435-2HG in tumors compared with normal samples showed that MIR4435-2HG was highly expressed in GC tissues (**Figure 2B**).

### Overexpression of MIR4435-2HG significantly promoted the proliferation of gastric cancer cells

**Figure 3:**
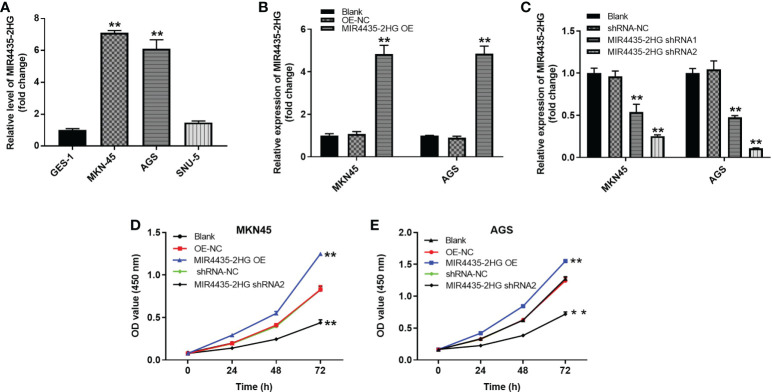
MIR4435-2HG expression results. **(A)** Expression of MIR4435-2HG in GES-1, MKN45, AGS and SNU-5 cells by RT-Qpcr. **(B)** MKN45 or AGS cells were transfected with MIR4435-2HG OE. Then, the expression of MIR4435-2HG in GC cells was detected by RT-qPCR. **(C)** MKN45 or AGS cells were transfected with MIR4435-2HG shRNA1 or shRNA2. Then, the expression of MIR4435-2HG in GC cells was detected by RT-qPCR. **(D, E)** The viability of gastric cancer cells was tested by CCK8 assay. **P < 0.01 compared with control (OE-NC; shRNA-NC).

The expression of MIR4435-2HG in various cells was detected using the RT-qPCR method. The results showed that MIR4435-2HG expression was significantly higher in MKN-45 and AGS cells than in GES-1 cells (**Figure 3A**). In addition, MIR4435-2HG OE significantly upregulated MIR4435-2HG expression in MKN-45 and AGS cells (**Figure 3B**) but was suppressed in the presence of MIR4435-2HG shRNA (**Figure 3C**), and GC cells were more sensitive to MIR4435-2HG shRNA2. MIR4435-2HG shRNA2 was chosen as the subject for the follow-up study. High MIR4435-2HG expression could promote the proliferation of MKN-45 and AGS cells (**Figures 3D, E**). In summary, overexpression of MIR4435-2HG significantly promoted the proliferation of GC cells.

### Overexpression of MIR4435-2HG increases the migratory ability of GC cells

**Figure 4:**
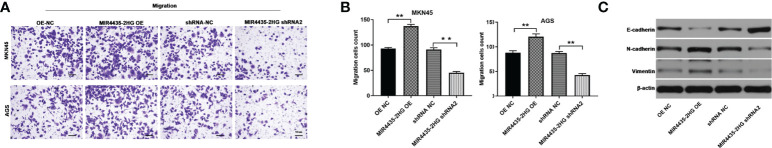
Overexpression of MIR4435-2HG increased the migration of gastric cancer cells. **(A, B)** The migration of gastric cancer cells was measured by the transwell assay. **(C)** The protein levels of E-cadherin, N-cadherin, and vimentin in GC cells were detected by western blot. **P < 0.01 compared with the control group.

Cell migration was examined using the transwell assay, and the results showed that overexpression of MIR4435-2HG significantly increased the migration of GC cells, whereas downregulation of MIR4435-2HG inhibited the migration of GC cells (**Figures 4A, B**). In addition, the expressions of N-cadherin and vimentin in GC cells were significantly upregulated by MIR4435-2HG overexpression but downregulated in the presence of MIR4435-2HG shRNA. In contrast, silencing of MIR4435-2HG significantly upregulated the E-cadherin protein level, whereas upregulation of MIR4435-2HG had an inhibitory effect on E-cadherin (**Figure 4C**). In conclusion, overexpression of MIR4435-2HG increased the migration of GC cells by promoting the EMT process.

### Results of exosome extraction and identification

**Figure 5:**
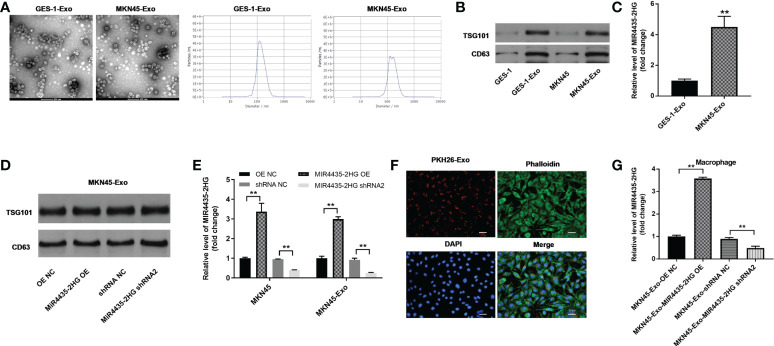
Results of exosome extraction and identification. **(A)** The separation efficiency of exosomes was examined by TEM. The particle sizes of exosomes were measured by NTA. **(B)** The expressions of TSG101 and CD63 in GES-1, MKN45 cells or exosomes derived from GES-1 or MKN45 cells were detected by WB. **(C)** The levels of MIR4435-2HG in exosomes derived from GES-1 or MKN45 cells were measured by RT-qPCR. **(D)** The expression of TSG101 in exosomes derived from MKN45 cells was detected by western blot. **(E)** The expression of MIR4435-2HG in MKN45 or exosomes derived from MKN45 cells was detected by RT-qPCR. **(F)** THP-1 cells were treated with 100 ng/ml of PMA and co-cultured with MKN45 cell-derived exosomes, and the location of exosomes was observed by immunofluorescence staining. **(G)** Macrophages were co-cultured with MKN45-Exo-OE NC, MKN45-Exo-MIR4435-2HG OE, MKN45-Exo-shRNA NC, or MKN45-Exo-MIR4435-2HG shRNA2. Then, the expression of MIR4435-2HG in macrophages was detected by RT-qPCR. **P < 0.01 compared with the control group.

Round particles with diameters of 30 to 150 nm were observed by TEM, and nanoparticle tracking analysis (NTA) yielded a size distribution close to that of TEM (**Figure 5A**). The expression of exosomal proteins (CD63 and TSG101) was significantly higher in GC cell exosomes compared with GC cells (**Figure 5B**). The expression of MIR4435-2HG in MKN45-Exo was significantly higher than that of GES-1-Exo (**Figure 5C**), whereas MIR4435-2HG overexpression or knockdown in MKN45 cell exosomes had a limited effect on exosomal protein levels (**Figure 5D**). MIR4435-2HG OE significantly upregulated MIR4435-2HG expression in MKN45 cells and MKN45 cell exosomes, and MIR4435-2HG shRNA2 significantly suppressed its expression (**Figure 5E**). Tumor-derived exosomes labeled with fluorescent PKH26 were internalized by unstained macrophages when co-cultured with macrophages (**Figure 5F**). Exosomes from MKN45 cells delivered MIR4435-2HG to PMA-treated THP-1 cells (**Figure 5G**). Exosomes were successfully isolated from GC cells.

### Overexpression of exosomal MIR4435-2HG promotes M2 polarization in macrophages

**Figure 6:**
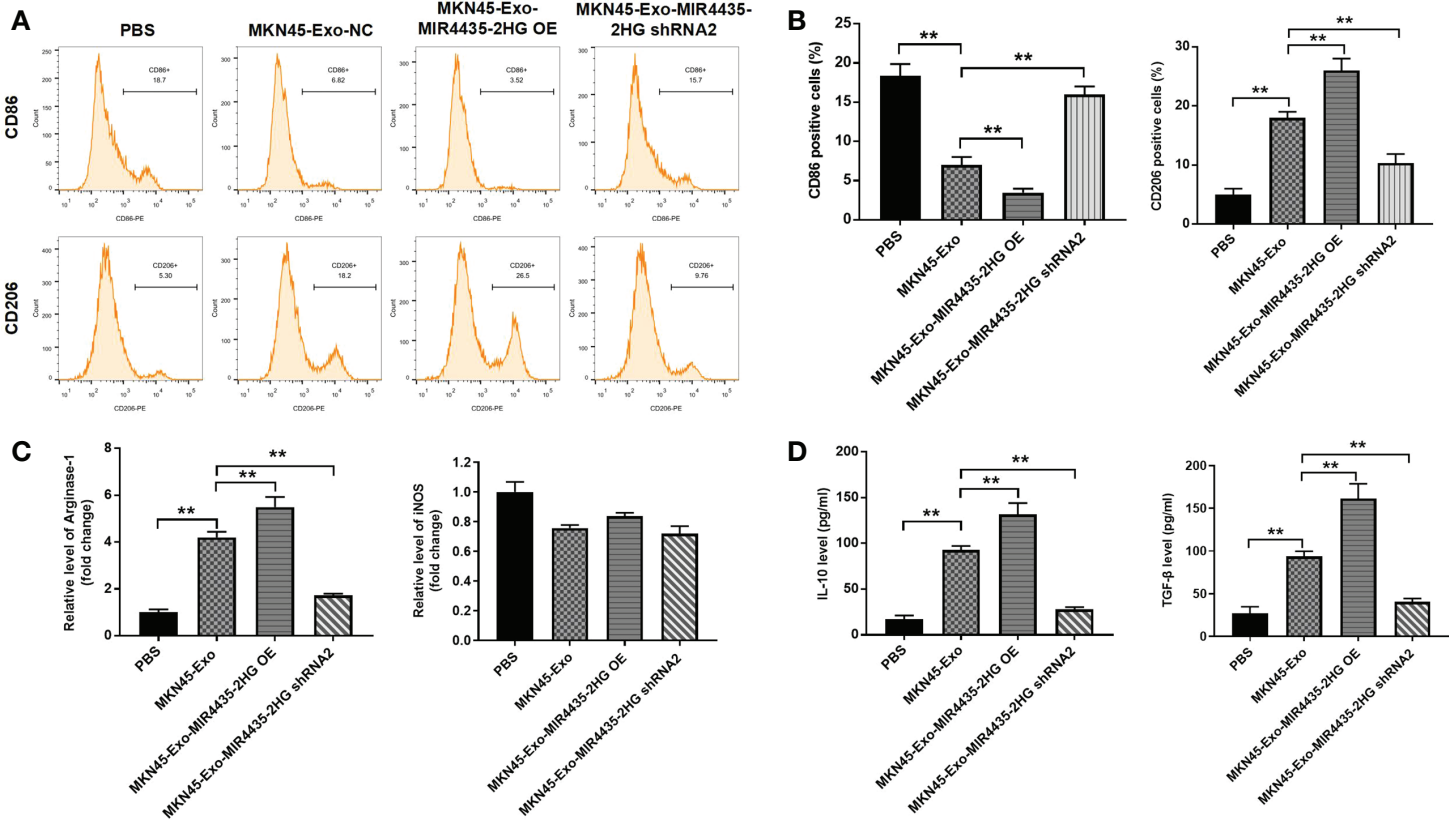
Exosome MIR4435-2HG promotes M2 polarization in macrophages. **(A, B)** Macrophages were co-cultured with MKN45-Exo-NC, MKN45-Exo-MIR4435-2HG OE, or MKN45-Exo-MIR4435-2HG shRNA2 for 24 h, and then the distribution rate of CD86 or CD206 in macrophages was detected by flow cytometry. **(C)** The levels of Arginase-1 and iNOS in macrophages were tested by RT-qPCR. **(D)** The levels of TGF-β and IL-10 in supernatants of macrophages were detected by ELISA. **P < 0.01.

To find the immunomodulatory effect of MKN45 cell-derived exosomes on macrophages, THP-1 cells were treated with 50 μg/ml of exosomes excreted from MKN45 cells. The distribution of CD86 (M1 phenotype marker) in THP-1 cells was significantly reduce, and THP-1 cells and macrophages exhibited the CD86low/CD206 high phenotype when cells were incubated with exosomes containing high levels of MIR4435-2HG. Additionally, exosomes from THP-1 cells significantly upregulated the distribution rate of CD206 (M2 marker) in THP-1 cells, which was partially reversed by exosomes downregulated with MIR4435-2HG (**Figures 6A, B**). Exosomes of MKN45 origin significantly upregulated arginase-1 levels, whereas exosomes downregulated with MIR4435-2HG reversed the effect of exosomes from MKN45 cells, and the exosomes had a limited effect on iNOS expression (**Figure 6C**). The expression of M2 markers (IL-10 and TGF-β) was significantly increased in macrophages incubated with MKN45 cell-derived exosomes compared to the PBS group, which was further exacerbated by MIR4435-2HG upregulated exosomes, and conversely, MIR4435-2HG downregulated exosomes partially reversed the effect of MKN45 cell-derived exosomes (**Figure 6D**). In summary, overexpression of exosomal MIR4435-2HG promotes M2 polarization in macrophages.

### Exosome-induced M2 macrophages promote the migration of GC cells by facilitating the EMT process

**Figure 7:**
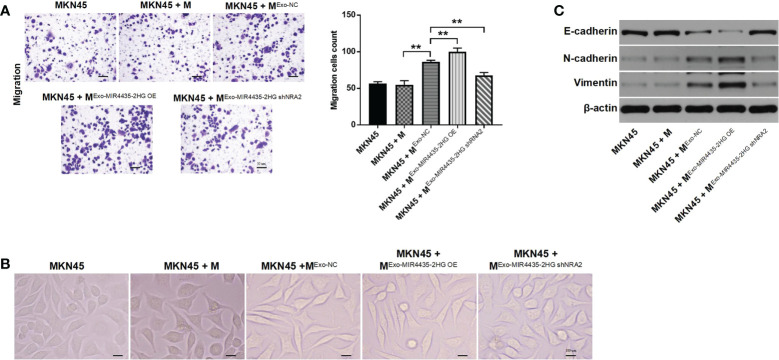
Exosome-induced M2 macrophages promote the migration of gastric cancer cells by inhibiting the EMT process. **(A)** MKN45 cells were co-cultured with macrophages, ExoNC-treated macrophages, Exo-MIR4435-2HGOE-treated macrophages, and Exo-MIR4435-2HGshRNA2-treated macrophages for 24 h, and the migration of MKN45 cells was detected by the transwell method. **(B)** The morphology of MKN45 cells was observed under a microscope. **(C)** The E-cadherin, N-cadherin, and vimentin protein levels in macrophages were detected by western blot. **P < 0.01.

Tumor cell-derived exosome-induced M2 macrophages significantly increased the migration of GC cells. Meanwhile, MIR4435-2HG upregulated exosomes aggravated this phenomenon, whereas MIR4435-2HG downregulated exosomes reversed it (**Figure 7A**). MKN45 cells exhibited a spindle-shaped morphology when co-cultured with M2 macrophages, which was reversed by MIR4435-2HG downregulated exosomes (**Figure 7B**). N-cadherin and vimentin expression was significantly increased in MKN45 cells after co-culturing with exosome-induced M2 macrophages, whereas MIR4435-2HG downregulated exosomes caused significant downregulation of N-cadherin and vimentin. The opposite data were obtained on E-cadherin expression (**Figure 7C**). In conclusion, exosome-induced M2 macrophages promote the migration of GC cells by increasing the EMT process.

### MIR4435-2HG in exosomes promotes M2 polarization of macrophages by regulating Jagged1/Notch and JAK1/STAT3 axes

**Figure 8:**
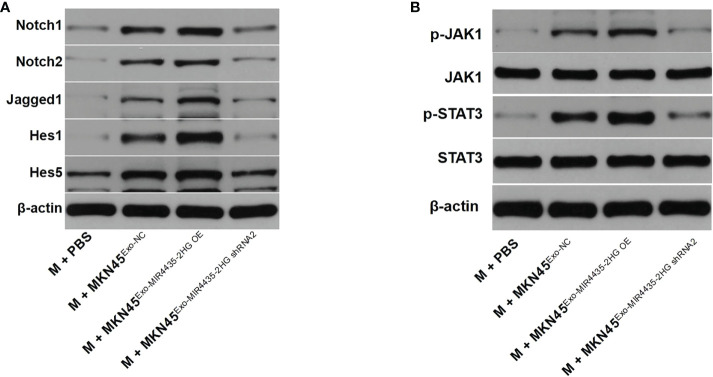
Exosomes from MKN45 cells promote M2 polarization in macrophages by regulating the Jagged1/Notch and JAK1/STAT3 axes. **(A)** The Jagged1, Notch1, Notch2, Hes1, and Hes5 protein levels in macrophages were detected by WB. **(B)** WB detection of p-JAK1, JAK1, p-STAT3, and STAT3 protein levels in macrophages.

Western blot results showed that exosomes from MKN45 cells significantly upregulated the protein levels of Notch1, Notch2, Jagged1, Hes1, and Hes5, whereas MIR4435-2HG downregulated exosomes partially inhibited the effect of exosomes on these proteins (**Figure 8A**). When MIR4435-2HG was downregulated in exosomes, its induction of the JAK1/STAT3 signaling pathway in macrophages was inhibited (**Figure 8B**). Taken together, exosomes from MKN45 cells promote M2 polarization in macrophages by regulating the Jagged1/Notch and JAK1/STAT3 axes.

### Upregulation of MIR4435-2HG in exosomes significantly promotes tumor growth in GC

**Figure 9:**
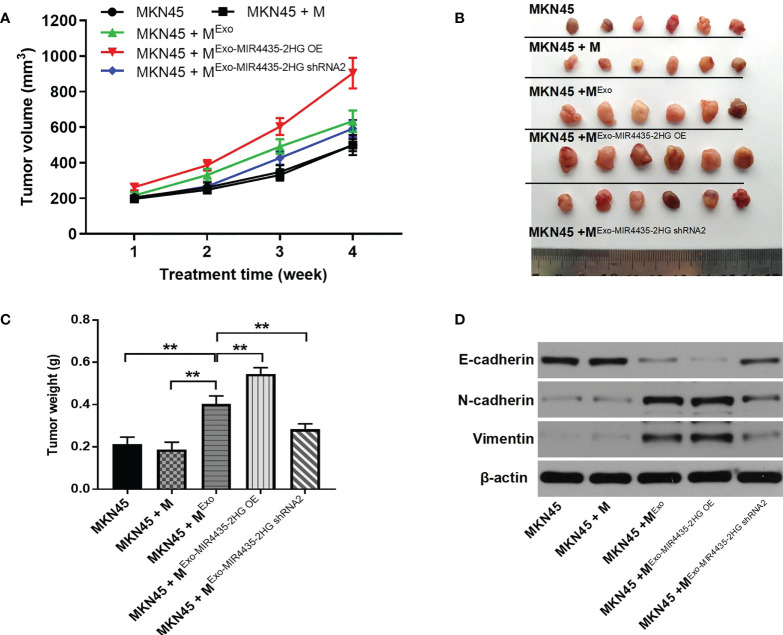
M2 m acrophages induced by exosomes significantly promotes the tumor growth of gastric cancer. **(A)** Tumor volumes of nude mice were tested. **(B)** Tumor tissues of nude mice were pictured. **(C)** Tumor weights of nude mice were measured. **(D)** The expression of E-cadherin, Ncadherin, and vimentin in tissues of nude mice were detected by WB. **P < 0.01 compared with MKN45.

Results from xenograft mouse models showed that exosomes from MKN45 cells significantly increased tumor size in nude mice, which was partially reversed by exosomes downregulated by MIR4435-2HG (**Figures 9A, B**). Exosomes from MKN45 cells greatly increased tumor weight in nude mice, which was partially reversed by exosomes downregulated by the MIR4435-2HG phenomenon (**Figure 9C**). MKN45 cell-derived exosomes significantly upregulated N-cadherin and vimentin expression in mouse tissues, whereas MIR4435-2HG downregulated exosomes reversed this phenomenon. Additionally, MKN45 cell-derived exosomes inhibited E-cadherin expression in nude mice, whereas MIR4435- 2HG OE upregulated exosomes further enhanced the role of exosomes (**Figure 9D**). Taken together, MIR4435-2HG upregulation in exosomes induced M2 polarization in macrophages, which significantly promoted the growth of GC tumors.

## Discussion

RNA interference is a phenomenon that can reverse the silencing of any gene, and clinical delivery materials are generally selected to transport siRNA to sites of action in target tissue cells (20). Exosomes, also known as signalosomes, can be expressed on exosomal membranes or packaged through ligands and adhesion molecules, thereby performing signaling (21). In this study, we focused on the role and potential value of MIR4435-2HG in GC, starting from the exosomes of GC cells.

The results showed that MIR4435-2HG upregulation could promote tumorigenesis in GC, and a previous study found that MIR4435-2HG could promote tumorigenesis in GC cells (22), which was consistent with our findings. In addition, we found that exosomal delivery of MIR4435-2HG from GC cells promotes the polarization of M2 macrophages, which in turn promotes GC development, and we speculate that exosomal MIR4435-2HG might act as an oncogene in GC and can be considered a marker of cancer tumorigenesis. Some studies have found that MIR4435-2HG is associated with various cancers, such as cervical cancer, GC, and renal clear cell carcinoma (23), which is consistent with our speculation. We also found that exosomes might increase migration of GC cells *in vitro* and *in vivo* when they induce selective activation of macrophages toward the M2 phenotype, and we hypothesized that macrophage M2 polarization might promote gastric carcinogenesis. One study found that macrophage M2 polarization can induce colorectal carcinogenesis by secreting CXCL13 (24). In addition, exosomes can be considered important mediators of the tumor microenvironment, and they can act as important messengers to regulate the cross-talk between different cells.

Jagged1 (JAG1) is an important Notch ligand, and JAG1/Notch signaling controls oncogenic processes in multiple cell types and is related to poor clinical prognosis (25). We found that exosomes from MKN45 cells can promote M2 polarization in macrophages by regulating the Jagged1/Notch or JAK1/STAT3 axis.

The JAK1/STAT3 axis is associated with phosphate metabolism, phosphorylation, and nuclear accumulation of STAT3 when JAK1 expression is upregulated (26), and the JAK1/STAT3 signaling pathway has been found to promote M2 polarization in macrophages (27), which is similar to our findings. The JAK1/STAT3 signaling pathway might be an M2 macrophage polarization-mediating factor.

In conclusion, we found that MKN45 cell-derived exosomes could induce M2 macrophage polarization and promote GC tumorigenesis; therefore, MIR4435-2HG could be used as a biomarker for GC and a new target for GC therapy.

## Data availability statement

The original contributions presented in the study are included in the article/supplementary material. Further inquiries can be directed to the corresponding author.

## Author contributions

CL: Conceptualization, Resources, Original Draft. ZC: Conceptualization, Resources, Review and Editing. JG: Conceptualization, Resources, Review and Editing. TT: Methodology, Original Draft, Review and Editing. LZ: Methodology, Original Draft, Review and Editing GZ: Methodology, Resources, Review and Editing. DZ: Validation, Resources, Supervision. CS: Validation, Original Draft, Supervision. LG: Validation, Original Draft, Project administration. TF: Formal analysis, Original Draft, Funding acquisition.

## Funding

National Natural Science Foundation of China (82002477).

## Conflict of interest

The authors declare that the research was conducted in the absence of any commercial or financial relationships that could be construed as a potential conflict of interest.

## Publisher’s note

All claims expressed in this article are solely those of the authors and do not necessarily represent those of their affiliated organizations, or those of the publisher, the editors and the reviewers. Any product that may be evaluated in this article, or claim that may be made by its manufacturer, is not guaranteed or endorsed by the publisher.
